# Pressure Drives Rapid Burst‐Like Coordinated Cellular Motion from 3D Cancer Aggregates

**DOI:** 10.1002/advs.202104808

**Published:** 2022-01-07

**Authors:** Swetha Raghuraman, Ann‐Sophie Schubert, Stephan Bröker, Alejandro Jurado, Annika Müller, Matthias Brandt, Bart E. Vos, Arne D. Hofemeier, Fatemeh Abbasi, Martin Stehling, Raphael Wittkowski, Johanna Ivaska, Timo Betz

**Affiliations:** ^1^ Institute of Cell Biology ZMBE University of Münster Von‐Esmarch‐Straße 56 D‐48149 Münster Germany; ^2^ Third Physical Institute University of Göttingen Friedrich‐Hund‐Platz 1 D‐37077 Göttingen Germany; ^3^ Institute of Theoretical Physics Center for Soft Nanoscience University of Münster Busso‐Peus‐Str. 10 D‐48149 Münster Germany; ^4^ Max Planck Institute for Molecular Biomedicine Röntgenstraße 20 D‐48149 Münster Germany; ^5^ Turku Biosience Centre University of Turku and Åbo Akademi University Turku FI‐20520 Finland; ^6^ Department of Life Technologies University of Turku Turku FI‐20520 Finland

**Keywords:** cancer invasion, cellular swelling, collectivity, coordinated cell motion, pressure

## Abstract

A key behavior observed during morphogenesis, wound healing, and cancer invasion is that of collective and coordinated cellular motion. Hence, understanding the different aspects of such coordinated migration is fundamental for describing and treating cancer and other pathological defects. In general, individual cells exert forces on their environment in order to move, and collective motion is coordinated by cell–cell adhesion‐based forces. However, this notion ignores other mechanisms that encourage cellular movement, such as pressure differences. Here, using model tumors, it is found that increased pressure drove coordinated cellular motion independent of cell–cell adhesion by triggering cell swelling in a soft extracellular matrix (ECM). In the resulting phenotype, a rapid burst‐like stream of cervical cancer cells emerged from 3D aggregates embedded in soft collagen matrices (0.5 mg mL^−1^). This fluid‐like pushing mechanism, recorded within 8 h after embedding, shows high cell velocities and super‐diffusive motion. Because the swelling in this model system critically depends on integrin‐mediated cell–ECM adhesions and cellular contractility, the swelling is likely triggered by unsustained mechanotransduction, providing new evidence that pressure‐driven effects must be considered to more completely understand the mechanical forces involved in cell and tissue movement as well as invasion.

## Graphical abstract

Coordinated bursts are a novel migration phenomenon where cancer cells escape from a model tumor into a soft extra‐cellular matrix made up of collagen. The interaction between the in‐homogeneous collagen and the tumor model via integrins leads to a series of dynamic effects such as cellular swelling, rise in pressure, and super‐diffusive coordinated bursts of cells into the ECM within 12 h.

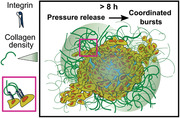

## 1 Introduction

The phenotypes that arise during cell migration are strongly influenced by the mechanical interplay between cells but also the extra‐cellular matrix (ECM) contributes vastly to the phenotypes of cell migration.^[^ ^1^, ^2^ ^]^ While migrating independently or within solid tissues, cells constantly experience shear forces, compression, tension, and hydrostatic as well as osmotic pressures.^[^ ^3^, ^4^, ^5^, ^6^, ^7^ ^]^ Because mechanical homeostasis ensures a complete force balance in tissues, single cells are not often observed to move on their own. However, this balance is broken when a migrating cell or a group of migrating cells need to generate well‐orchestrated forces. Over the past decades, our understanding of cell migration has been boosted by a series of ground‐breaking experiments that allowed not only for classifying different kinds of collective and single‐cell migration, both in vitro and in vivo, but also for identifying a series of key molecular players that drive this motion.^[^ ^1^, ^4^, ^8^, ^9^ ^]^ Yet, our knowledge of forces and force generation lags far behind, with most insights suggesting that cell migration is driven by actin polymerization in mesenchymal‐like migration, and myosin‐driven contractility in amoeboid‐like migration.^[^ ^10^, ^11^ ^]^ Although it is well established and thoroughly studied, this notion inherently excludes other mechanisms that may drive cellular motion, such as pressure. In particular, this view stands in contrast to a series of hallmark experiments in the field of tumor biophysics that have demonstrated that pressure and pressure distribution within solid tumors can be critical for proliferation, migration and protein expression.^[^ ^12^, ^13^, ^14^, ^15^, ^16^, ^17^, ^18^ ^]^ Regarding its effects on migration, pressure has been observed to be crucial for breaching the basement membrane during the formation of the anterior posterior axis in mouse embryo development.^[^ ^19^ ^]^ Overall, these findings show that pressure is not just a simple consequence of cell proliferation in confined geometries, but it may also play a pivotal role in cellular functions, development and tumor progression. Although it may seem intuitive that pressure relaxation could lead to tissue and cellular motion resembling collective or active cell migration, it has not yet been addressed as a realistic interdependent mechanism involving pressure and signaling required for cell migration.

One reason for this might be because in experimentally attractive in vitro systems, such as cancer aggregates embedded in ECM matrices, pressure‐driven cell movement has not been observed. Typically, cell invasion from cancer aggregates into an ECM depends on classical migration mechanisms, where cells actively pull on the ECM. In such situations, any potential pressure increase in the aggregates would be released by these pulling forces. In fact, cells at the periphery of in vitro aggregates sense and respond to the ECM via integrins, upon which cells undergo changes in morphology, polarity, and contractile forces; these changes in contractile forces are quantified as collective cell‐generated ECM pulling forces that enable the movement of the ECM toward the aggregate, facilitating migration.^[^ ^20^, ^21^, ^22^, ^23^ ^]^

To overcome the problem that pressure‐driven cell and tissue movement is not accessible in vitro, here we introduce a model that produces the phenotype of cellular bursts emerging from cancer aggregates embedded in ECM with a low concentration of collagen. With this model system, we demonstrate that such pressure‐induced cell dispersal represents a coordinated behavior, as depicted by increasing migration velocity correlations. Further, we show that these pressure‐driven outbursts involve cell–ECM adhesions and acto‐myosin contraction‐based signaling that are required for an increase in the cellular volume, eventually leading to a rise in pressure. Surprisingly, this correlated motion during the outbursts remains independent of cell–cell adhesions, suggesting that this process is distinct from classical collective migration. Using a computer simulation based on the initial experimental conditions, we fully recapitulate the experimental result, further supporting the finding that pressure‐driven coordinated cell dispersal is a novel mechanical phenotype of cellular movement.

## 2 Results

### 2.1 Rapid Outbursts Triggered by a Low Density Collagen Micro‐Environment

**Figure 1:**
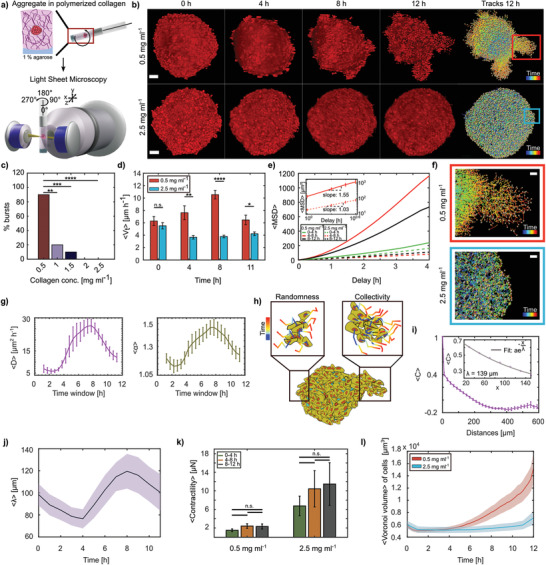
HeLa aggregates collectively burst in 0.5 mg mL^−1^ collagen matrix. a) Schematic representation of aggregate preparation and imaging process. b) Over 8 h, aggregates in 0.5 mg mL^−1^ collagen (LCC; top) burst into the matrix, whereas aggregates embedded in 2.5 mg mL^−1^ collagen (HCC; bottom) did not. Nuclei tracks at 12 h from each condition are shown, scale bars = 50 µm. c) Percentage of bursts in collagen concentrations 0.5 to 2.5 mg mL^−1^ (*N* = 10 each scenario). d) Comparison of average absolute cell velocities 〈*V*_t_〉 within aggregates embedded in LCC & HCC at different time points (*N* = 10 each, standard error of the mean (s.e.m.)). e) Average MSD for three time windows (*N* = 10 each). Inset shows the MSD in log scale between 4–8 h for LCC and HCC, with their corresponding slopes (s.e.m.). f) Zoomed‐in view of the bursts (red box) and noninvasive (blue box) regions from (b) (scale bars = 20 µm). g) Averages of diffusivity (*D*) and powerlaw exponent α obtained by fitting a linear function onto the log of window MSDs (*N* = 10 each, s.e.m.). h) Representation of both the early randomness and later collectivity in motion as observed in LCC outbursts. i) Velocity correlation 〈*C*〉 binned over distances (*N* = 10, bin size = 20 µm). Exponential decay function fit on distances up to 150 µm at 8 h (inset). j) Mean velocity correlation length acquired over time (median smoothed, *N* = 10, s.e.m.). k) Contractile stresses exerted by aggregates in LCC and HCC on their ECM (*N* = 5 each, s.e.m.). l) Mean Voronoi volume of cells within aggregate acquired over time (*N* = 10 each, s.e.m.).

To investigate pressure‐driven collective migration we use cancer cell aggregates as standard model systems which allows us to systematically study the interaction between 3D simple tissues and well‐controlled ECM microenvironments^[^ ^20^ ^]^ (**Figure** **1**a). Exploiting light sheet‐based 3D microscopy, we followed the motion of individual HeLa cells within aggregates by tracking their fluorescently marked nuclei via histone H2B‐mCherry or H2B‐RFP. HeLa tumor models embedded in 2.5 mg mL^−1^ collagen I (higher collagen concentration, HCC) did not show any shape changes over time but simply grew due to proliferation (Figure 1b). However, by merely reducing the collagen concentration to 0.5 mg mL^−1^ (lower collagen concentration, LCC) without any other changes to the cell culture or sample preparation, we observed a drastic migration phenotype marked by rapid cell outbursts starting between 6–8 h post embedding (hpe), in which a large amount of cells were expelled into the surrounding ECM (Figure 1b,f, Videos S1 and S2, Supporting Information). To test whether the HCC environment simply delays the outbursts, we waited up to a week, but no outbursts were observed. (Figure S1, Supporting Information). Thus, the drastic burst phenotype suggests a critical phenomenon, as a threshold concentration exists. To better understand how the phenotype changes based on different collagen concentrations, we embedded aggregates in a series of different concentrations ranging from 0.5 to 2.5 mg mL^−1^ with a step size of 0.5 mg mL^−1^ (Figure 1c). Surprisingly, increasing collagen concentrations did not produce a continuous decay of the burst phenomenon but instead a rapid loss.

This behavior suggests that a threshold process drives the bursts. To understand whether the biophysical properties of the collagen also undergo such a drastic change and, hence, may be responsible for the observed phenotype, we characterized the collagen ECM. However, we found that, while collagen density and ligand density increased with collagen concentration, as expected, the fiber length and total fiber number only changed marginally (Figure S2a– e, Supporting Information). This suggests that for the polymerization conditions we used, the collagen underwent an increase in bundling at higher concentrations, which was indeed found when we determined the fiber diameter. Consistent with this, the stiffness measurements also confirmed that the storage component of the collagen shear modulus increased up to threefold in the HCC compared to the LCC (Figure S2f, Supporting Information). To simplify the following investigation of the observed outbursts, we focused on the two extreme cases (0.5 and 2.5 mg mL^−1^).

### 2.2 Outbursts are Super‐Diffusive and Show Increased Velocity Correlation, Reminiscent of Collective Cell Migration

As the collagen properties did not give a simple explanation for the observed phenotype we decided to precisely quantify the motion and tracks of the cells within the aggregate and during the outbursts. The quantitative analysis of the cell tracks revealed that during the first hour after embedding, the overall velocity of the cell motion within the aggregate was similar in the two different collagen concentrations, but between 4–8 hpe we found a drastically higher mean absolute cell velocity within aggregates embedded in the LCC matrices than in the HCC (Figure 1d). Additionally, upon visual inspection, the obtained phenotype showed a more directional motion during the bursts. Indeed, detailed analysis of the mean squared displacement (MSD) demonstrated that both conditions followed a power law behavior 〈*x*(τ)^2^〉 = 6 D τ^α^, where *D* is the diffusion constant and τ the lag‐time. However, while cells in the HCC behaved diffusively, as quantified by an exponent of α_HCC_ = 1.03, in the burst phase we saw super‐diffusive motion (α_LCC_ = 1.55), suggesting ballistic‐like behavior (Figure 1e,h). To understand whether the LCC condition had an immediate or rather delayed effect on this directional movement, we created a time‐resolved MSD by a 2.5‐h sliding window (Figure 1g and Figure S3, Supporting Information). Both the time evolution of the diffusion coefficient and the power law exponent showed time dependence, with a peak at 7–8 hpe, which is consistent with the observed morphological change during the bursts. Surprisingly, the power law exponent showed a complex switching behavior. After an initial instance of slight super‐diffusive behavior (α_LCC, 0_ = 1.2), it first tended briefly toward normal diffusion before drastically increasing to its peak value and finally decreasing again, ending close to the initial value. This suggests that the coordination of the cell movement changed several times within a rather short time span of only 12 h. To further confirm this, we checked the correlation length of the velocity correlation function (Figure 1h,i), which is a typical cue for collective migration as seen from previous studies.^[^ ^24^, ^25^ ^]^ Indeed, the correlation length λ showed a similar behavior as the power law exponent, with an initial decrease that was followed by a monotonic increase up to a peak value of about 120 µm at 8 hpe, before it again decayed (Figure 1j). These rapid and fundamental changes in migration behavior are intriguing, as they are typically associated with differential gene expression, for example, in the context of epithelial–mesenchymal transition.^[^ ^26^ ^]^

As the observed switching back and forth between random and collective migration seems at odds with biology, we instead considered a physical explanation for the observed bursts. Therefore, we checked the forces exerted by the cells on the collagen matrix. We found that the forces did not significantly change over time. However, when comparing the total force in the different collagen concentrations, we confirmed the already known mechanosensing mechanism since cells exerted higher forces in the stiffer HCC (Figure 1k).^[^ ^27^ ^]^ The finding that forces are constant over time is puzzling, as it shows that rapid and collective cell migration during the bursts is not accompanied by a significant increase in traction forces applied on the ECM. To further investigate what factors are driving the outbursts, we checked the volume of the cells in the aggregate based on a Voronoi tessellation that uses the nucleus as a proxy for the cell position. As presented in Figure 1l, we saw an impressive and rapid increase in cell volume within aggregates in the LCC condition, whereas in the HCC the volume remained unchanged over the same time span.

### 2.3 Changes in Migration Modes are Accompanied by Aggregate Shape Changes and a Volume Increase

**Figure 2:**
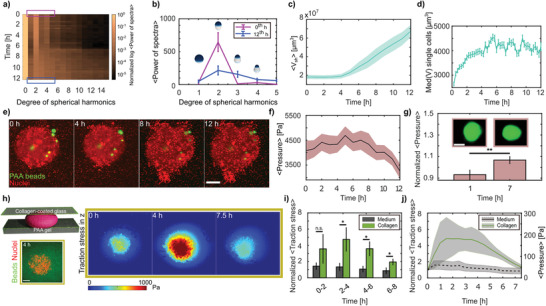
Cell volume increase in LCC leads to increased internal pressure. a) Temporal contribution of degrees 0–15 of spherical harmonics expansions on aggregate surfaces, color coded by the normalized (by degree 0) log of average power spectra (*N* = 10). b) Mean spherical harmonics spectra for 0 and 12 h, over the expansion degrees 1–5 (*N* = 10, s.e.m.). Scheme represents zero‐order shape contributions. c) Mean volume 〈*V*_sh_〉 increase as determined from the spherical harmonics (median smoothed, *N* = 10, s.e.m.). d) Median (Med) HeLa single cells volume *V* in LCC (*n* = 1694–2124 for each time point, *N* = 3, s.e.m.). e) Data showing polyacrylamide (PAA) elastic beads (green) randomly distributed within aggregates (scale bar = 100 µm). f) Mean anisotropic pressure recorded by PAA beads (median smoothed). g) Anisotropic pressures normalized by the average pressure at the binned hours represented (bin size = 1 h). Insets depict deformation of a bead at 0 and 7 h (scale bar = 10 µm). Statistical test was done on log‐normalized data. h) Schematic of sandwich experiment to confine aggregates and perform traction force microscopy (left, bottom: zoomed‐in view of single plane image at the interface between PAA gel and the aggregate, scale bar = 100 µm). Indicative data showing average traction stress in Pascals (Pa) in the *z* direction over time (right). Pushing (red) forces indicated. i) Normalized (by time 0) average traction stresses in *z* direction of aggregates confined with collagen embedding as compared to only medium. Statistical tests were done on log‐normalized data. j) Normalized (by time 0) and relative average aggregate pressure by the sandwich method. For (f) and (g) *n* = 35–51, s.e.m.; (i) and (j) *N* = 6 (collagen), 5 (medium), s.e.m.

To further understand both the changes in the motility characteristics and the volume change in the LCC condition, we developed a second approach to quantify the aggregate shape and volume using a mathematical decomposition into spherical harmonics (SHs). SHs allow one to develop any radial surface function into a series of shapes with increasing complexity, where the relative contribution of each shape is quantified by a single parameter (**Figure** **2**a,b). As expected, the aggregate volume obtained from the SHs closely followed the time evolution that was observed for the Voronoi tessellation (Figure 2c). However, a close inspection of the different degrees and their relative contributions gave an additional surprise. While in the first 2 h, the actual shape of the aggregate resembled an oblate, as quantified by the second mode of the SHs, we saw a rapid decrease of this mode within 5 h, followed by a drastic increase of the higher modes marking the burst phase (Figure 2a,b) starting at 7 hpe. Hence, up until 4 hpe the aggregates changed their initial oblate shape toward a spherical shape without any volume change. Then, a massive volume increase set in, which we hypothesize effectively pushed cells outward in the observed bursts. This is consistent with the observed super‐diffusive motion and higher correlation, as the cells moved in a coordinated way. To test whether individual cells embedded in the LCC condition would also show the observed volume increase, we dispersed isolated HeLa cells in the LCC condition and measured the change in volume. Consistent with the aggregate, we found a drastic increase in volume immediately after seeding (Figure 2d). Interestingly, the volume increase within the aggregate started later, which suggests that in the tumor model embedded in LCC condition, contact with other cells partially stabilizes the cell volume.

### 2.4 Internal Aggregate Pressure Rises before Burst Phase

The observed lack of correlation between the ECM pulling forces and the outbursts suggests that the cells did not migrate out in a classical collective migration mechanism, but that the observed bursts were the collective effect of the cell volume increase. Such swelling may have led to a pressure increase inside the aggregate, similar to previous observations in mouse embryos and epithelial monolayers.^[^ ^5^, ^6^ ^]^ To confirm this volume‐increase‐based pressure‐rise hypothesis, we exploited recently introduced elastic hydrogel beads as force and pressure sensors and analyzed their deformation over the time course of the experiment (Figure 2e and Video S3, Supporting Information). Indeed, we found that the internal forces acting on the pressure sensors increased monotonically and showed a significant peak at 6–7 hpe, correlating with the onset of the outbursts (Figure 2f,g).

After verifying that the pressure inside the aggregate was increasing, we wondered whether this could also exert a consequential pushing force on the environment, which would directly prove that the cells were pushed out. To test this, we devised a modified 2.5D traction force microscopy approach,^[^ ^28^, ^29^ ^]^ where the aggregate was confined in a sandwich‐like fashion between a bottom polyacrylamide (PAA) gel of Young's modulus *E* ≈ 1800 Pa and a top layer of collagen I‐coated glass (Figure 2h), without compressing the aggregate. When the aggregates were surrounded by LCC, we could see a significant increase in pushing forces, marked by *z*‐traction stress at 2–6 hpe of up to ≈ 200 Pa, thereby deforming the bottom gel (Figure 2i,j and Figure S4, Supporting Information). As the stiffness of the LCC was only about 10 Pa (see ref. [30], and confirmed by rheometry, Figure S2f, Supporting Information), we did not expect that the aggregate would generate larger forces, as most cells started to move laterally in this situation (Video S4, Supporting Information). Nevertheless, these experiments confirmed that the pressure inside the aggregate increased in the LCC condition and that this rise caused the cells to generate a pushing force on the environment. Consistent with the lack of bursts in the absence of collagen and in the HCC situation, we did not see similar pushing forces in either of those situations (Figure 2i,j and Figure S4, Supporting Information). This further supports the hypothesis that during the outbursts, cells are being pushed by the pressure within the aggregates, which is induced by the cell swelling.

### 2.5 Coordinated Pressure‐Driven Bursts Depend on Cell–ECM Adhesion

**Figure 3:**
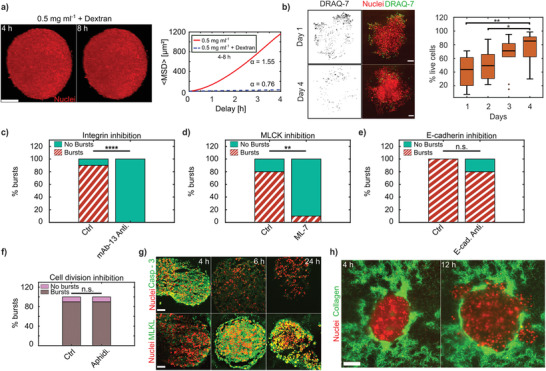
Coordinated pressure‐driven cell dispersal depends on cell–ECM interactions and is independent of cell–cell adhesion and proliferation. a) Dextran pressure of 18 kPa in LCC stops bursts from the aggregates, scale bar = 100 µm. 〈MSD〉 between 4–8 hpe displaying subdiffusive behavior in LCC + dextran (*N* = 1) and super‐diffusion in LCC (*N* = 10). b) HeLa H2B‐mCherry aggregates stained for DRAQ‐7 (green) on days 1 and 4 (left) post embedding. Percentage of cells alive from days 1 to 4 (*N* = 10 each). Medians indicated, scale bars = 100 µm. c) Percentage of bursts in control (Ctrl (*N* = 10), LCC) as compared to in the presence of anti‐integrin antibody (mAb‐13 Anti. (*N* = 10), 30 µg mL^−1^) to inhibit integrin‐based cell–ECM adhesions. Bursts were checked at 8 hpe. d) Percentage of bursts in control (Ctrl (*N* = 10), LCC) as compared with the presence of drug (ML‐7 (*N* = 10), 100 µm) to inhibit MLCK. e) Percentage of bursts in control (Ctrl (*N* = 3), LCC) as compared with the presence of anti‐E‐cadherin antibody G‐10 (E‐cad. Anti. (*N* = 10) to inhibit cell–cell adhesion, 10µg mL^−1^). f) Percentage bursts in control (Ctrl, *N* = 10) and with aphidicolin (Aphidi. *N* = 10, 5 µg mL^−1^). All scenarios in (d–f) were checked for bursts at day 1 post embedding. g) 12 µm cryosections of aggregates stained for caspase‐3 and MLKL antibodies at 4, 6, and 24 h, scale bars = 50 µm. h) Collagen pockets in LCC seen at 4 and 12 hpe, scale bar =100µm.

To further test the hypothesis that the cells are being pushed from the aggregate to release the pressure, we decided to artificially increase the pressure acting from the ECM side on the aggregate,^[^ ^31^ ^]^ thus reducing the differential pressure. The rationale for this experiment is that a pressure difference‐dependent effect can be suppressed by effectively abolishing the pressure difference. This was achieved by adding 100 mg mL^−1^ dextran 2 MDa to the ECM, which generates a compressive pressure of about 18 kPa,^[^ ^32^ ^]^ thereby exceeding the average pressure increase we measured within aggregates using the elastic beads. Such dextran concentrations are shown to be inert to cells^[^ ^32^ ^]^ and, hence, should only reduce or even inverse the pressure difference. Consistent with the pressure‐driven outgrowth hypothesis, removing the pressure difference in the LCC situation fully eradicated the outbursts within the same time scale (**Figure** **3**a and Figure S5a, Supporting Information). Interestingly, this counteracting surrounding pressure further reduced the cell motility and led to subdiffusive motion, which directly confirms that the cells were pressed together in a jamming‐like fashion (Figure 3a, right).

Although the pressure‐driven effect provides an excellent mechanical explanation for the observed cell bursts, it does not illuminate the underlying mechanism that triggered the cell swelling as the source of the observed pressure increase. Hence, to test whether cell–ECM adhesions were involved in triggering the outbursts in LCC, we administered an antibody mAb‐13 (30 µg mL^−1^) against inactive integrin beta‐1 which thus acts as an antagonist for the collagen binding by reducing the availability of active ECM‐bound integrins. The percentage of bursts was checked at 8 hpe (due to high turnover of integrins at the surface of cells), and we noticed that integrin‐inhibited aggregates failed to burst (Figure 3c). Motivated by this clear sign that integrin‐mediated cell–ECM interaction is at the core of the driving mechanism, we wondered whether the swelling was due to the chemical binding of integrin to collagen or, alternatively, due to a failure in establishing mechanotransduction. In a simple experiment to check this, we added inert, elastic hydrogel in the space between the collagen fibers. This was achieved by adding low melting agarose (LMA) to the collagen mixture in the LCC situation. Effectively, this increased the stiffness of the composite material while keeping the collagen concentration constant (LCC). We expected that the enhanced mechanical rigidity of the composite would then be recognized by the cells. Indeed, adding the mixture of LCC and LMA drastically reduced the bursts, consistent with the hypothesis that the soft ECM is required for the burst phenotypes (Figure S5c, Supporting Information). By only adding the LMA, no chemical activation of the integrins took place, and the cells did not interact with the environment; hence, no bursts occurred (Figure S5c, Supporting Information). In contrast, when mixing the collagen with other ECM proteins, such as fibronectin and laminin (Figure S5b, Supporting Information), the chances of burst occurrence remained high, thus fully confirming the pivotal role of cell–ECM adhesions for the burst phenotype.

### 2.6 Burst Motion is Distinct to Classical Cell Motility

The above findings suggest that the chemical activation of the integrins and the subsequent mechanical probing is required for the bursts. As cell shape and size are regulated by acto‐myosin contractility, we tested the outcome of inhibiting the myosin light chain kinase (MLCK) activity with the help of the drug ML‐7 (100 µm). Indeed, this drastically eliminated the bursts, however, to our big surprise, inhibition of ROCK or Rac‐1 via Y‐27632 and NSC23766 respectively did not reduce the outbursts (Figure S6a,b, Supporting Information). This suggests that MLCK‐activated contractility plays a vital role in the mechanical interplay between the soft LCC matrix and the outbursts (Figure 3d). Furthermore, it demonstrates that the outburst movement is different from classical integrin‐driven mesenchymal cell migration and that in the LCC condition, forces are generated by the volume increase. Hence cell motion in the bursts is driven by a process distinct from that of commonly described cell migration. While this may initially seem puzzling, it fits perfectly with the finding that forces are generated by the volume increase, that then pushes the cells into the surrounding ECM. To avoid confusion, it seems reasonable to simply talk about cellular motion instead of cell motility, which commonly implies the classical mesenchymal or amoeboid migration.

Furthermore, the observation of long‐range correlation in cellular velocity demonstrates collective behavior. As the force generation mechanism in the outbursts seems to differ from the forces at play in classical cell migration, we then wondered whether the mode of collective motion was also distinct from the classical situation. Collective migration is defined to depend on pulling forces transmitted between cells.^[^ ^2^ ^]^ Obviously, the here proposed pushing mechanism does not require pulling forces but instead relies on pushing forces that are independent of cell–cell adhesion. To test whether the observed coordinated motion depends on such adhesion, we used a cadherin antibody binding on the extracellular domain (Figure 3e) to suppress cell–cell adhesion. In line with the pressure‐driven model, we observed that the bursts occurred 80% of the time, even after reducing the cell–cell adhesions. This confirms that the pressure‐based coordinated motion mechanism is different from classical collective migration schemes.

In conclusion, these experiments show that the observed outbursts are driven by a new, yet‐undescribed mechanism that resembles collective migration but is different. Here, force generation is triggered by a drastic cellular volume increase, while long‐range movement correlation of movement is due to steric interactions where cells push each other, similar to a liquid flowing along a pressure difference.

### 2.7 Pressure‐Driven Bursts are Independent of Cell Proliferation and Correlate with Cell Death

The volume increase of the aggregate may be partially explained by the observed increases in individual cell volumes, but it might also be explained by proliferation. To identify the extent to which mitosis is involved in the observed outbursts, we arrested cells in S‐phase of the cell cycle by adding aphidicolin (5 µg mL^−1^). However, this did not reduce the burst phenotype (Figure 3f), suggesting that in our model system, proliferation is not essential for the coordinated bursts.

As suggested by the dependence of the burst phenotype on integrins and MLCK contraction, cell swelling may have resulted from unsustained mechanotransduction due to the soft environment in the LCC. As previous work has suggested that failed opposition to cytoskeletal tension can lead to cell death,^[^ ^33^, ^34^, ^35^ ^]^ we speculated that the tumor aggregates' exposure to the soft matrix (LCC) could also lead to massive cell death. To test this, we used the membrane‐permeable dead cell stain DRAQ‐7, which marks nuclei in the case of plasma membrane rupture. Indeed, we found that 1 day post embedding, a high number of cell nuclei stained positive for DRAQ‐7 (Figure 3b), demonstrating increased cell death. Furthermore, cell death increased in the regions of the outbursts, suggesting that bursts are related to reduced cell survival. Since DRAQ‐7 is a classical necrosis marker, we further wanted to confirm whether apoptosis possibly preceded the cell death, as reported previously.^[^ ^36^ ^]^ Staining against the apoptosis marker caspase‐3 was highest at 4 hpe, whereas staining for the mixed lineage kinase domain‐like pseudokinase (MLKL, for necrosis) starkly increased later, between 6–24 hpe (Figure 3g). Thus, these markers establish the series of events leading to cell death during the burst phase. Interestingly, when inspecting the live aggregates 4 days after bursts, the DRAQ‐7 staining was largely absent, indicating that the overall cell viability had recovered, even though the environment remained the same. We quantified this recovery by recording the percentage of live cells as a function of days (Figure 3b and Figure S8, Supporting Information) and confirmed that already at 2 days post embedding, the viability increased and recovered to almost 90% living cells after 4 days.

### 2.8 Outburst Regions are Directed by Limited Mechanical Resistance Due to Collagen In‐Homogeneities

So far, we have demonstrated that the bursts are pressure‐driven, independent of cell proliferation and cell–cell adhesion, and generated by cell swelling that is triggered by the LCC. However, these findings do not explain why localized outbursts occur instead of a homogeneous aggregate expansion. Motivated by the sandwich experiments, where we saw lateral motion of the cells while the aggregate was pushing into the PAA substrate, we hypothesized that in‐homogeneities in the LCC structure could facilitate an expansion into regions with the least mechanical resistance. Indeed, when imaging the collagen after embedding the aggregate, we saw large variability in spatial concentration (Figure 3h), potentially due to the low overall collagen concentration or as an outcome of the embedding process (Experimental Section). Consistent with the hypothesis of bursts into regions of least resistance, we predicted that the bursts would occur precisely in the directions of reduced collagen presence.

**Figure 4:**
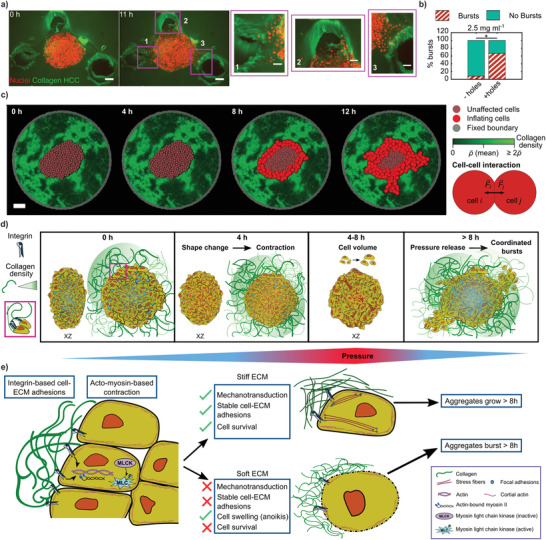
Less local mechanical resistance of the ECM can drive bursts in LCC or HCC. a) Holes were introduced into HCC at 0 h, into which cells burst out from the aggregate when followed over 12 h. Shown is the maximum intensity projection (scale bar = 100 µm). Zoomed insets show the cells bursting in the direction of the three holes (scale bar = 50 µm). b) Percentage of bursts in HCC with (+) and without (‐) holes introduced. *N* = 12 each. c) Simulation results for the time evolution of a cancer aggregate. The initial distribution of cancer cells and collagen was chosen analogous to the experimental data (scale bar = 100 µm). d) Sketch representing series of events over 0–8 h triggering coordinated pressure‐driven burst‐like dispersal of cells. [0 h] Cells at the periphery of oblate aggregates attach to the LCC via integrins (see legend and zoomed inset). Internal pressure remains low. [4 h] The ECM attachment sculpts a shape change. Pressure starts to be disturbed. [4–8 h] Volume of cells increases within aggregates, raising the internal pressure. From [8 h] onward, aggregates are unable to sustain the pressure increase and expel cells collectively in the directions of least mechanical resistance (green gradient (legend) depicts collagen density (in‐homogeneity in LCC)). e) Sketch of signaling events that precede the bursts. In the initial hours, once cells attach to the collagen fibers, the cell–ECM adhesions trigger the phosphorylation, and thus, activation of the myosin light chain. This leads to the binding of myosin II to actin cables, initiating the acto‐myosin‐based contraction of cells. The succeeding events depend on the rigidity of the ECM. In the presence of a stiff ECM (HCC), the cells receive the necessary mechanical feedback from stable cell– ECM adhesions that sustain a force balance and promote cell survival. Conversely, when the cells are surrounded by soft ECM (LCC), poor mechanotransduction stems from unstable cell–ECM adhesions, as the cells contract while pulling on the ECM. Such a failed force balance leads to drastic consequences such as cell swelling, possibly due to anoikis, that ultimately causes cell death. As a result, in the stiff ECM scenario, the aggregates grow beyond 8 h unperturbed, and within the same time span in the soft ECM case, the aggregates burst into the regions that provide the least mechanical resistance, thereby releasing the pressure (refer to (d)) in the aggregates.

Moreover, if this prediction were to hold true, then we could speculate that as long as the local mechanical resistance was sufficiently low to trigger the volume increase and allow the pressure release, then the concentration of the collagen should not matter. Hence, we should be able to rescue the burst phenotype even in the HCC situation, which so far had not shown outbursts. To check this, we embedded aggregates in 2.5 mg mL^−1^ and introduced local mechanical weaknesses by punching holes in three different locations ≈ 100–200 µm away from the surface of aggregates (**Figure** **4**a and Video S6, Supporting Information). Of note, the cells were always in contact with the 2.5 mg mL^−1^ collagen, and only the mechanical resistance was decreased due to the introduced void. The cells had no information about the position of the voids, despite the local mechanical resistance to pulling forces. As predicted by the poor mechanotransduction hypothesis, we did indeed recover the burst phenotype even in the HCC situation. This suggests that bursts are triggered not only by the chemical interaction between the cells and the ECM but also by the state of the ECM's mechanical resistance.

Furthermore, in these experiments, the cell bursts were mostly directed toward the introduced holes/pockets, indicating that they preferred to burst into the regions of least mechanical resistance (Figure 4a insets). When quantified further in comparison to the HCC scenario without the holes (Figure 4b), we noticed that the number of bursts increased by more than fivefold, providing evidence that collagen in‐homogeneities are sufficient to trigger the signaling cue for the outburst phenotype. These results confirm the hypothesis that the burst localization simply reflects the local mechanical properties.

To further test the hypothesis that swelling drives the bursts while in‐homogeneity of the collagen directs it, we simulated the experimental situation. To this end, we modeled the aggregate as a cluster of spheres embedded in an in‐homogeneous environment, mimicking the experimental situation (Figure 4c). When applying the observed volume increase to the individual cells as observed in the experiments (Figures S7 and S9, Supporting Information), the simulations correctly reproduced the coordinated outbursts. This demonstrates that the model proposed here (Figure 4d,e) can explain the observations, even when using a highly simplified situation of interacting spheres and an in‐homogeneous ECM.

## 3 Conclusion

Here, we reported a new type of pressure‐driven coordinated cellular motion that is initiated by the contraction of cells in a low collagen concentration environment (Figure 4d,e). The acto‐myosin‐based contraction that is driven by the presence of the ECM explains the observed initial collective and super‐diffusive motion occurring as the aggregate, as a whole, changes from an oblate to a more spherical shape. In this process, we suspect that in the presence of a soft ECM (LCC), the integrin‐based cell–ECM adhesions and the acto‐myosin‐based signaling tend not to be able to sustain the mechanical interplay necessary for the cells, due to LCC condition's insufficient mechanical feedback in response to cellular pulling forces. Such an unsustained mechanotransduction results in cellular swelling which leads to a sharp pressure increase within the aggregate. The pressure difference with the environment subsequently pushes the cells, which results in the observed outbursts. Similar to anoikis,^[^ ^37^ ^]^ the low collagen concentration scenario leads to massive cell death, with bursts of cells streaming out in the direction of the least mechanical resistance. The preference of cancer cells to invade with high velocities into pre‐existing tracks has also been observed in vivo in mice melanoma tumors, wherein cells widen these interfacial tissue tracks by passively pushing and expanding the space provided.^[^ ^38^ ^]^ A simple explanation to such pushing can be speculated to be driven by pressure within tumors that propels the cells as described in the in vitro scenario depicted here. This model is consistent with all our observations and suggests a new mechanism of large‐scale coordinated cellular transport that echoes collective migration but is inherently different, as it is independent of cell–cell adhesions and is driven by pressure. While this mechanism contradicts the standard paradigm for collective migration, which requires cell–cell adhesions to migrate, but still shows a correlated migration behavior during the outbursts, it reflects a simple and almost intuitive outcome of pressure release. Although here we report a special situation for low collagen concentration matrices, the general pressure‐driven burst mechanism might be highly relevant in the context of cancer cell invasion. Primary tumors are often encapsulated by a basement membrane that constrains the growing tumor. Upon increased proliferation, the pressure inside the tumor is known to increase. Additionally, the basement membrane may rupture either by simple mechanical tension or due to active degradation by the cancer cells. Given that being able to disrupt the basement membrane is a major roadblock for cancer cells aiming to invade the ECM, getting pushed by the pressure within a solid tumor might be a highly efficient way for cells to exit the solid stresses they experience within these tissues via cell outbursts. Once into the environment, either as single cells or collectively, they may face additional challenges, but the cells' initial propulsion at high velocity may be enough to thrust them further into regions that provide least resistance, thus potentially producing drastic consequences while promoting metastasis.

## 4 Experimental Section

### Cell Culture and Aggregate Preparation

HeLa cervical cancer cell lines (wildtype, a kind gift from Roland Wedlich‐Söldner) were stably transduced to express H2B‐mCherry, H2B‐mCherry along with Life‐Act‐GFP or, H2B‐mCherry and MyrPalm‐GFP, via lentiviral transduction. HeLa cell expressing H2B‐RFP and α‐tubulin‐GFP was a kind gift from Matthieu Piel. All cells were cultured using high glucose Dulbecco's modified Eagle's medium (DMEM, Capricorn) supplemented with 10% (v/v) fetal bovine serum (FBS, Sigma‐Aldrich) and a 1% (v/v) penicillin–streptomycin solution (Gibco). Cells were stored at 37 °C in a humidified atmosphere with 5% CO_2_ and were split on reaching a confluency of >60%. Split ratios were either 1:5, 1:10, or 1:20.

Cancer aggregates were prepared in a 48‐well plate (Greiner Bio‐one) as previously described.^[^ ^20^ ^]^ Plates coated with 150 µL of 1% ultra‐pure agarose (Invitrogen) in each well were cooled for 30 min prior to adding 1 mL of cell suspension containing 2000–2500 cells. The aggregates were collected between days 2–4 and imaged.

### Collagen Polymerization

Collagen concentrations of either 0.5 or 2.5 mg mL^−1^ were prepared using a mixture of 10× phosphate‐buffered saline (PBS, Sigma‐Aldrich) (diluted to 1× in the final volume prepared), rat tail collagen I (Corning, stock: 3.77 mg mL^−1^), and cell culture medium. The polymerization was activated by adding 1 m NaOH to attain a pH of 7.5. All solutions used were stored in 4 °C before and kept on ice under sterile conditions while preparing the polymerization mix. For collagen concentrations 1, 1.5, 2 mg mL^−1^, the polymerization procedure was the same. CO_2_‐independent medium supplemented with 10% (v/v) FBS (Sigma‐Aldrich) and a 1% (v/v) penicillin–streptomycin solution (Gibco) was used instead of DMEM for experiments performed with either light sheet or spinning disk confocal microscopes.

In order to mark or label the collagen fibers, a collagen‐binding adhesion protein CNA‐35 labeled with eGFP (CNA35‐eGFP, stock: 8.2 mg mL^−1^, a kind gift from Gijsje Koenderink) was used at 1:20 of the final collagen concentration for either 0.5 or 2.5 mg mL^−1^ as required.

### Biophysical Characterization of Collagen

Fiber radius, collagen, and ligand density: Labeled collagen gel (Experimental Section) of increasing concentrations from 0.5 to 2.5 mg mL^−1^ in steps of 0.5 mg mL^−1^ were spread on a glass slide with a 12 mm round glass cover slip (VWR). Slides containing 0.5 and 1 mg mL^−1^ gels were polymerized first for 15 min at 37 °C. All samples were then sealed (Biotium, CoverGrip) to avoid evaporation and further polymerized at room temperature (22–24 °C) for 1 h prior to imaging. After 1 h, images were captured with a 60× water immersion objective of a spinning disk confocal system (CSU‐W1 Yokogawa, Intelligent Imaging Innovations Inc.) via the Slidebook 6 software (3i) equipped with an inverted microscope (Nikon Eclipse Ti‐E) and a CMOS camera (Orca‐flash4.0v2, Hamamatsu Photonics K.K.), with stack size of 30 µm and step size of 1 µm. These images were then further processed using CT‐FIRE^[^ ^39^ ^]^ to retrieve the fiber lengths and total number of filaments from the different collagen concentrations. To obtain the collagen density, the corrected fluorescence intensity (after offset subtraction) of the CNA35‐eGFP that binds to the collagen fibers was used as a proxy for the collagen density. Here, the fluorescence images were thresholded by setting the background to 0. As expected, the resulting fluorescence linearly scales with the used collagen concentrations. As the collagen density is defined as the total volume occupied by collagen in the sample *V*_c_ divided by the measurement volume *V*_M_, the offset corrected fluorescence *F*_c_ can be used to estimate the relative changes in *V*_c_ by: *F*_c_∝*V*_c_. Here, *V*_c_ = π〈*R*〉^2^〈*N*〉〈*L*〉 is defined, where 〈*R*〉 is the average fiber radius, and 〈*N*〉, 〈*L*〉 are the average number and length of detected filaments, respectively. Therefore, the relative change in fiber radius was estimated as: $\left\langle R\right\rangle \propto \sqrt{\frac{\left\langle F_{c}\right\rangle }{\left\langle N\rangle \langle L\right\rangle }}$. Using the same argumentation, the relative change in ligand density was determined as proportional to the surface area of the collagen fibers. Hence ligand density ρ_L_∝*A*_c_, where the total surface area of the collagen fibers was estimated as *A*_c_ = 2π〈*R*〉〈*L*〉〈*N*〉. Hence, using the above approximation for the fiber radius, the desired result for the ligand density was finally obtained: $\rho_{L}\propto \sqrt{\left\langle F_{c}\right\rangle \left\langle L\right\rangle \left\langle N\right\rangle }$. An error propagation (relative error) for the estimated fiber radius and ligand density was done based on the relation $\sigma {}_{\text{rel}}=\sqrt{{\left(\frac{\sigma {}_{N}}{\left\langle N\right\rangle }\right)}^{2}+{\left(\frac{\sigma {}_{L}}{\left\langle L\right\rangle }\right)}^{2}+{\left(\frac{\sigma_{F_{c}}}{\left\langle F_{c}\right\rangle }\right)}^{2}}$. The absolute errors were then deducted by $\sigma_{R}=\sigma_{\text{rel}}\left|\left\langle R\right\rangle \right|$ and $\sigma {}_{\rho_{L}}=\sigma {}_{\text{rel}}\left|\left\langle \rho_{L}\right\rangle \right|$.

**Rheology**: Unlabeled collagen concentrations of 0.5 and 2.5 mg mL^−1^ were prepared as mentioned before (Experimental Section). The MCR‐301 Anton Paar bulk rheometer was used to measure the viscoelastic properties of both concentrations by compressing the gel with a 25 mm diameter cone–plate at 37 °C. Mineral oil was added around the cone–plate edge to avoid evaporation during measurement. A constant shear strain with a strain rate ($\dot{\gamma }$) of 0.5% at 1 Hz for 15–30 min was applied to measure the *G*′ (storage modulus) and *G*″ (loss modulus).

**Collagen Contractility**: To obtain the forces that the aggregate exerts on its environment, a recently published procedure^[^ ^23^ ^]^ was adapted to the aggregate burst system. Briefly, fluorescently labeled particles (100 nm diameter) embedded in the collagen matrix were tracked over time to reconstruct the local deformation of the hydrogel. Using macro‐rheology, the nonlinear response of the collagen gel was measured and used to create a lookup table that relates the observed deformation to the exerted pressure. Averaging over all directions, a mean contractility (pressure × surface area, μ N) and s.e.m. were obtained.

### Aggregate Sample Preparation and Multi‐View Imaging

To image the aggregate at sub‐cellular resolution a dual illumination light sheet microscope (Zeiss Z.1, 20× objective, N.A. 1.0) was used. Fluorinated ethylene propylene (FEP) capillaries (Proliquid, dimensions: 1.6 mm × 2.4 mm, width: 0.4 mm) along with Teflon plungers (Zeiss, size 3) suitable for incorporating into the microscope's sample holder were used. Once the cells aggregated on day 2 or 3, they were first removed from the 48‐well plate and transferred to a Petri dish (92 mm × 16 mm, Sarstedt). 100 µL of collagen polymerization mixture was introduced into the Petri dish at room temperature (22–24 °C). Aggregates were then transferred into this collagen droplet with minimal medium to avoid dilution of the droplet. Finally, the aggregates were pulled into the FEP capillary via the suction force provided by the plunger. The capillary was then repeatedly rotated horizontally by hand, holding the part where the aggregate was suspended in the polymerizing collagen until it was stably centered (15–20 min) within collagen fibers. 1% ultra‐pure agarose (Invitrogen) was then plugged at the bottom to prevent collagen from seeping out of the capillaries over time. The sample capillaries were then introduced into the sample chamber of the light sheet microscope filled with CO_2_‐independent medium supplemented with 10% (v/v) FBS (Sigma‐Aldrich) and a 1% (v/v) penicillin–streptomycin solution (Gibco) equilibrated to 37 °C, 30 min prior to imaging.

Nuclei of cells from aggregates within polymerized collagen with concentration of 0.5 mg mL^−1^ (LCC) were imaged with a 15 min time interval, and those of 2.5 mg mL^−1^ (HCC) were imaged with both 15 min (*N* = 5) and 1 h (*N* = 5) time intervals. Multi‐views of the samples were acquired at four different angles (multiples of 90°). Each view comprised *z*‐stacks in the range of 600–800 µm, separated by an optimal distance of 2 µm. The lateral resolution for image stacks was in the range 0.45–0.56 µm and the light sheet thickness was ≈ 6.47 µm. Fluorescent beads (505 nm, Invitrogen; 561 nm, Micromod) of sub‐cellular resolution were embedded in the collagen in order to register the different views. Registration was done using the “Multiview Reconstruction”^[^ ^40^, ^41^ ^]^ plug‐in from Fiji.^[^ ^42^ ^]^ The point spread function of the microscope could be estimated from these registration beads using the plug‐in, and images were deconvolved and downsampled 2× or 3× for further analysis.

### Single Cells Sample Preparation and Imaging

The preparation was done immediately after passaging HeLa cells (stably expressing H2B‐mCherry and LifeAct‐GFP) using culture media. Cell pellets were resuspended in 1 mL CO_2_‐independent medium. LCC polymerization mixture volume of 100 µL consisting of 80 000 cells was prepared by adjusting the volume of the medium (for collagen) with respect to the volume of cells added in the mixture. The sample mix was not vortexed to prevent any damage to cells. Once the pH was adjusted to 7.5, the sample mix was pulled into the FEP capillary and rotated until the collagen polymerized (similar to aggregate preparation) and imaged with the light sheet microscope.

### Cell Detection and Tracking in Aggregates

Nuclei marked for H2B‐mCherry or H2B‐RFP were tracked via the “Autoregressive Motion” algorithm in Imaris 9.3.0 through the “Spot detection and tracking” method. *XY* diameters were estimated from the “Slice” view and thresholds were set for the minimum intensity and quality of detection in order to filter for noise spots detected. All further analyses of tracks were done using MATLAB R2016a. Nuclei that were unable to be segmented because their intensities did not match the threshold criteria were omitted, and track positions were interpolated for missed links (typically 1–2).

### Track Analysis: Absolute Velocity

The absolute velocity $|V_{i}|$ for every particle *i* in 3D (*x*, *y*, *z*) was calculated as $\sqrt{{\left(V_{x,i}\right)}^{2}+{\left(V_{y,i}\right)}^{2}+{\left(V_{z,i}\right)}^{2}}$ over time. Averages and s.e.m. were then acquired for every time point.

**MSD**: The MSD of all particles over the time delays was calculated using a previously described method.^[^ ^43^ ^]^ The time window‐based MSD was an adapted version of the method, where it was only applied on the tracks within the respective time windows, given a window size of ten time points (total 49 time points (12 h) with 15 min time interval). A linear function was fitted to the logarithmic data of the equation 〈*x*(τ)^2^〉 = 6*D*τ^α^ in the moving time window (2.5 h) to attain the exponent α and the diffusivity constant *D* for a given delay τ.

**Voronoi Volume of Cells within Aggregates**: Based on tracked nuclei positions, a MATLAB function “N–D Voronoi diagram” was applied to retrieve an estimate for the cell borders. Next, all cell borders per time point were fit by an ellipsoid (ellipsoid fit, https://www.mathworks.com/matlabcentral/fileexchange/24693‐ellipsoid‐fit, MATLAB Central File Exchange) to extract an approximation for the cell volume.

**Velocity Correlation**: The velocity vectors of all particles *i* per time point *t* were correlated with every other particle *j* and normalized by the square of the mean absolute velocities of *i* and *j*, leading to the correlation function

(1) $$\begin{array}{cc} C_{\mathrm{ij}} & =\frac{V_{i}\cdot V_{j}}{{\left(\frac{|V_{i}\left|+\right|V_{j}|}{2}\right)}^{2}}\; \end{array}$$

*C*_*ij*_ was binned over the distances x, between particles i,j (bin size = 20 µm), and the averages per bin were acquired for each time point. Next, 〈*C*(*x*)〉 up to a binned distance of 150 µm was fit by an exponential function $f\left(x\right)=a\;e^{-b\;x}$, where the correlation length is defined as $\lambda =\frac{1}{b}$.

### Spherical Harmonics for Shape Change and Aggregate Volume

Spherical harmonics are a complete set of orthogonal functions defined on the unit sphere and are extracted using the Python toolbox shtools.^[^ ^44^ ^]^ To determine the shape and volume changes of the aggregates over time, their surfaces were described using the expansion

(2) $$\begin{array}{cc} r(\theta ,\phi ) & =\sum_{l=0}^{l_{\max}}\sum_{m=-l}^{l}c_{lm}Y_{lm}\left(\theta ,\phi \right) \end{array}$$

where *r* denotes the distance of a point on the surface from the center of mass of the aggregate, θ and ϕ are the spherical coordinate angles, *c*_*lm*_ the spherical harmonic coefficients, *Y*_*lm*_ the spherical harmonics, *l* and *m* their degree and order, respectively, and *l*_max_ the maximum degree of the expansion.

The enclosed volumes of these surfaces were determined by the integral

(3) $$\begin{array}{cc} V_{\text{sh}}= & \int_{0}^{2\pi }d\phi \int_{0}^{\pi }d\theta \int_{0}^{r(\theta ,\phi )}dr\;r{\left(\theta ,\phi \right)}^{2}\sin\theta \; \end{array}$$

The higher the limit of the sum *l*_max_, the finer the details of a surface that can be described by the spherical harmonics. Due to this, on the raw data a variable expansion degree was used as the shape of the aggregates evolved, starting with typically *l*_max_ = 4 for the oblate shape at earlier time points and reaching up to *l*_max_ = 15 for the intricate shapes occurring at later time points as a consequence of the bursts.

It was observed that such a variation in the spherical harmonic expansion degrees on raw data over time gave rise to offsets/artifacts in the volume detection, that had to be corrected. Assuming that drastic changes in volume do not occur at short time scales, and understanding the error source due to the expansion degree changes, the volume was readjusted by adding the difference caused by the offset to the consecutive time points. To verify whether the detected volumes were influenced by the raw experimental data, a fixed expansion degree of *l*_max_ = 15 was used and the volumes were determined based on aggregate surfaces estimated from tracked nuclei positions. The results were in close agreement with those from the raw data.

### Volume Measurement of Single Cells

To measure the volume of single HeLa cells in LCC, stably expressing H2B‐mCherry and LifeAct‐GFP were used. The cell shapes via LifeAct‐GFP were 3D segmented using CellProfiler 3.1.9^[^ ^45^ ^]^ on downsampled data (2.78 µm pixel^−1^). A global “Otsu” threshold was applied to segregate pixel intensities to background or foreground. This was followed by a standard “Watershed” algorithm, and segmented objects were measured for shape volume. Exported measurements were extracted and represented using MATLAB R2016a.

### Elastic Beads as Pressure Sensors

A custom‐made^[^ ^46^ ^]^ water‐in‐oil emulsion was used to prepare the inert PAA beads of Young's modulus E=3.9±0.9 kPa by injecting the water‐based bead mix in oil dissolved in n‐Hexane (Supelco). The PAA beads were fluorescently labeled (Atto 488 NHS‐Ester, Atto‐Tec) several times until sufficient fluorescent signal was achieved. HeLa H2B‐mCherry aggregates were prepared (Experimental Section) such that each well of the 48‐well plate contained ≈ 30 PAA beads. Aggregates with randomly incorporated elastic beads were imaged in the same way as mentioned above (Experimental Section).

To calculate the pressure applied on the elastic beads by the cells within aggregates, the main force dipole deforming the bead surfaces was first retrived using spherical harmonics expansions. For this, it was sufficient to use the spherical harmonic components *Y*_00_ and *Y*_20_ as

(4) $$\begin{array}{cc} & r{\left(\theta ,\phi \right)}_{\mathrm{bead}}=\frac{c_{00}}{\sqrt{4\pi }}+c_{20}\sqrt{\frac{5}{16\pi }}\;\left(3\cos^{2}\theta -1\right) \end{array}$$

where the first term corresponds to the radius of the beads by $r_{0}=\frac{c_{00}}{\sqrt{4\pi }}$ and the second term describes the major uniaxial deformation of a sphere. The surfaces of the beads were expanded in the zeroth and second degrees of the spherical harmonics using the above function, and the surfaces were rotated in such a way that the *c*_20_ coefficient was minimized, aligning then the main axis of compression with the *z*‐axis. Using this expression as an ansatz to tackle the elastic problem on a sphere as expressed in the Navier–Cauchy equation, the force dipole acting on the bead was obtained

(5) $$\begin{array}{c} F=\frac{G}{2}c_{00}c_{20}\sqrt{5}\left(\frac{N_{r}\left(\nu \right)}{4}+\frac{3N_{\theta }\left(\nu \right)}{2}\right)e_{z} \end{array}$$

with $N_{r}\left(\nu \right)=\frac{2\nu }{1-2\nu }+\frac{1}{2(2-3\nu )(1-2\nu )}+\frac{1}{2(2-3\nu )}$ and $N_{\theta }\left(\nu \right)=\frac{1}{2}-\frac{1}{4-6\nu }$, and ν and *G* being the Poisson ratio and shear modulus of the PAA beads, respectively.

The pressure was then estimated from the relation $P=\frac{F}{A}$, where *A* is taken as the surface of the bead

(6) $$\begin{array}{c} P=G\;c_{20}\;\frac{\sqrt{5}}{2\;c_{00}}\left(\frac{N_{r}\left(\nu \right)}{4}+\frac{3N_{\theta }\left(\nu \right)}{2}\right)\; \end{array}$$

### Measuring the Internal Pressure by Confining Aggregates

HeLa H2B‐mCherry day 2 aggregates were equilibrated for 1 h in CO_2_‐independent medium containing 10% (v/v) FBS (Sigma‐Aldrich) and a 1% (v/v) penicillin‐streptomycin solution (Gibco) at 37 °C prior to using them for sandwich experiments. The sandwich consisted of a bottom layer of PAA gel (*E* ≈ 1800 Pa) and a top layer of a 12 mm round glass cover slip (VWR).

Bottom layer: A 35 mm glass‐bottom dish (Greiner Bio‐one) was first thoroughly cleaned with 0.1 m NaOH, silanized, and washed twice. The dishes were then treated with 25% glutaraldehyde for 30 min. A pre‐gel mixture of 40% acrylamide and 2% bis‐acrylamide in a 2:1 ratio, along with 4 µL of 99% acrylic acid was prepared and diluted with 65% PBS (Sigma‐Aldrich) to achieve the required Young's modulus (E≈1800 Pa). 10 µL fluorescent beads (505 nm, Invitrogen) were added to the pre‐gel mix for traction force microscopy.^[^ ^28^, ^29^ ^]^ The free radical polymerization was triggered by adding 5 µL ammonium persulfate and 1.5 µL tetramethylethylenediamine. A 5 µL drop of the final pre‐gel mix was added to the center of the glass‐bottom dish and spread evenly by placing a 12 mm round cover slip on top for 20 min. The cover slip was then slid away from the gel after adding 65% PBS to the dish. For all experiments the PBS was aspirated completely and the gel was air‐dried for 10 min before use.

Top layer: The top layer of the confinement was coated overnight with 50 µg mL^−1^ collagen I (Corning, stock: 4.88 mg mL^−1^) in 0.2 m acetic acid containing fluorescent beads (≈1%).

100 µL of the collagen solution (LCC or HCC) prepared was then added to a Petri dish (92 × 16 mm Sarstedt), to which 2–3 aggregates were introduced, avoiding dilution with medium. The aggregates were then transferred one by one with ≈ 20 µL collagen volume to the PAA gel. The top and bottom layer preparations were the same for both LCC and HCC experiments.

Confinement and imaging: Images of the aggregates were taken using a spinning disk confocal system (CSU‐W1 Yokogawa, Intelligent Imaging Innovations Inc.) via the Slidebook 6 software (3i) equipped with an inverted microscope (Nikon Eclipse Ti‐E) and a CMOS camera (Orca‐flash4.0v2, Hamamatsu Photonics K.K.). The microscope contains a custom‐built heating chamber, which was maintained at 37 °C during imaging. In order to confine the aggregates within a sandwich‐like model, the top layer was adhered with the help of korasilon paste (Kurt Obermeier GmbH) to a movable top.

The top layer was moved down to the upper periphery of the aggregates, such that they were only confined and not compressed. Once the positions of the aggregates were stabilized, 3 mL of CO_2_‐independent medium was added. *z*‐stacks with intervals of 0.5 or 1 µm were acquired from the bottom to the top layer, with a time interval of 30 min for ≈ 8 h. For control experiments, no‐collagen was involved for the bottom or the top layers. Reference images for traction force microscopy in LCC and no‐collagen scenarios were acquired after the imaging once the top layer was moved away and the aggregates were dissociated from the bottom gel with the help of 10% sodium dodecyl sulfate. In the HCC scenario, reference images were taken before the top layer was moved down or when the aggregate still had not deformed the bottom gel.

### Applying External Pressure in LCC

0.5 mg mL^−1^ collagen was polymerized along with 100 mg mL^−1^ dextran 2 MDa (Dextran T2000, Pharmacosmos) to achieve a pressure of ≈ 18 kPa.^[^ ^32^ ^]^ Aggregates were then subject to this environment and imaged for 12 h using the light sheet microscope (Zeiss Z.1, 20× objective, N.A. 1.0).

### Chemical Reagents to Inhibit Aggregate Bursts

ML‐7 (Sigma‐Aldrich, I2764, 100 µm), mAB‐13, stock 0.67 mg mL^−1^, used at 30 µg mL^−1^), aphidicolin (Sigma‐Aldrich, A4487, 5 µg mL^−1^), Y‐27632 (Sigma‐Aldrich, Y0503, 50 µm), NSC23766 (Sigma‐Aldrich, SML0952, 100 µm) and E‐cadherin antibody (G‐10, SCBT, 10 µg mL^−1^) were used. Aggregates (from day 3) to be treated with all chemicals except for E‐cadherin antibody and NSC23766, were preincubated with the chemicals along with culture media for 1 h at 37 °C in a humidified atmosphere with 5% CO_2_. After an hour, chemical‐treated preincubated aggregates and E‐cadherin‐ or NSC23766‐untreated aggregates were embedded in FEP capillaries containing LCC along with the respective drug/chemical (*N* = 10 for each). Sample capillaries were immersed in 1 mL culture medium (with or without drug/chemical (control)) and stored at 37 °C in a humidified atmosphere with 5% CO_2_ overnight. After 24 h, bright‐field images were taken using an inverted microscope (Vert.A1 Zeiss, Axio). For the aggregates treated with mAB‐13, images were taken at 8 hpe.

### Quantifying Cell Survival

FEP capillaries consisting of HeLa (stably expressing H2B‐mCherry, or H2B‐mCherry and LifeAct‐GFP) aggregates that had undergone a burst‐like cell dispersion within 8 h of sample preparation (Experimental Section), were continued to be stored at 37 °C in a humidified atmosphere with 5% CO_2_ from days 1–4 (*N* = 10, for each day). After each day, the capillaries were removed and samples were pushed out carefully into a Petri dish (6015 mm, Greiner Bio‐one), after which 100 µL of culture medium was added on top of each of the aggregates (still in LCC) to prevent drying out. From here on, the samples were protected from light, and DRAQ‐7 (Thermofisher) was administered at 1:100 ratio to every sample droplet, and the Petri dish was stored at room temperature for 10 min. Spinning disk confocal images of the nuclei with lasers 561 nm and 647 nm (DRAQ‐7) were acquired with the Slidebook 6 software (3i) using an inverted microscope (Nikon Eclipse Ti‐E) equipped with a CSU‐W1 spinning disk head (Yokogawa) and a scientific CMOS camera (Prime BSI, Photometrics).

Nuclei were detected using Laplacian of Gaussian detection algorithm from Trackmate.^[^ ^47^ ^]^ The number of dead cells was subtracted from the total number of cells detected, to retrieve the % live cells.

### Cryosectioning and Immunostaining Aggregates

Capillaries consisting of HeLa H2B‐mCherry day 3 aggregates in 0.5 mg mL^−1^ collagen were removed from the medium at 4, 6, and 24 h and immersed in 1× PBS (three times) to wash. The capillaries were dipped into 4% paraformaldehyde 1 mL for 30 min. Next, capillaries were washed again three times with 1× PBS. To preserve the structure of the bursts in collagen, the capillaries were subjected to 500 µL optimal cutting temperature mounting medium (Sakura) for 1 h. They were then partially snap‐frozen with liquid nitrogen vapor. After 30 s at room temperature, the frozen samples were pushed out of the capillaries into a block of liquid optimal cutting temperature mounting medium and centered before being snap‐frozen completely. Samples were stored at −80 °C until further processing.

Frozen aggregates were sectioned, mounted as 12 µm sections and re‐hydrated. The samples were blocked for 1 h at room temperature using 1× PBS supplemented with 10% goat serum (Sigma‐Aldrich) and 0.2% Triton‐X‐100 (Carl Roth). Subsequently, the samples were incubated with the primary antibody (monoclonal mouse anti‐caspase‐3, 1:100, Santa Cruz, sc‐56053; monoclonal mouse anti‐MLKL, 1:100, Proteintech, 66675‐1‐Ig) diluted in blocking solution over night at 4 °C. After three washes with PBS, samples were incubated with the secondary antibody (polyclonal goat anti‐mouse IgG1, 1:500, ThermoFisher) diluted in blocking solution for 45 min at room temperature. Samples were finally washed with PBS three times, and confocal images were acquired with Slidebook 6 software (3i) using an inverted microscope (Nikon Eclipse Ti‐E) equipped with a CSU‐W1 spinning disk head (Yokogawa) and a scientific CMOS camera (Prime BSI, Photometrics).

### Triggering Bursts in HCC

HeLa aggregates (*N* = 10) were embedded in 2.5 mg mL^−1^ (HCC) collagen inside FEP capillaries (Experimental Section) and stored at 37 °C in a humidified atmosphere with 5% CO_2_ for 7 days to check for signs of bursts. As a control, aggregates (*N* = 10) were also embedded in 0.5 mg mL^−1^ (LCC) and stored similarly for a day. Bright‐field images were taken on day 7 for HCC and day 1 for LCC, and compared.

To trigger bursts in HCC, 4 mm diameter wells (6‐well ibidi flow chambers) were used for sample preparation. The bottoms of the wells were coated with 10–20 µL of 1% agarose. After cooling the wells for 15 min, 20 µL of freshly prepared collagen HCC was added, and the wells were then kept in 37 °C in a humidified atmosphere with 5% CO_2_ for 5–6 min. Next, the wells were taken out and aggregates were added at the center of the gel with no medium. The wells were again kept in 37 °C in a humidified atmosphere with 5% CO_2_, now for 1 min. This ensured the aggregates were stable with the collagen fibers around them. After 1 min, 20 µL of previously prepared HCC was added on top as a second layer, and the wells were again stored in 37 °C for 12 min. Next, holes were poked into the polymerized HCC with the help of a preparation needle (Omnilab) in three different locations, approximately close to the aggregate. In the control scenario, no holes were introduced into the sample. 40 µL medium was finally added to the wells and stored at 37 °C in a humidified atmosphere with 5% CO_2_ and checked for bursts the next day. In the case of a 11–12 h experiment, the confocal images were taken via Slidebook 6 software (3i) using an inverted microscope (Nikon Eclipse Ti‐E) equipped with a CSU‐W1 spinning disk head (Yokogawa) and a scientific CMOS camera (Prime BSI, Photometrics).

### ECM Dependency

Mix of ECM proteins: To prepare a mixture of ECMs, fibronectin (100 µg mL^−1^, kind gift from Lydia Sorokin) and laminin (100 µg mL^−1^, Sigma‐Aldrich) were mixed with 0.5 mg mL^−1^ collagen and polymerized together (Experimental Section). Aggregates were embedded in such a mixture and prepared in FEP capillaries as described above (Experimental Section). The capillaries were then stored at 37 °C in a humidified atmosphere with 5% CO_2_ for 24 h, at which point bright‐field images were taken to check for bursts.

Hydrogel‐based ECM: To observe the ECM dependency, aggregates were embedded in three different environments, a) only 0.4 % low melting agarose (Invitrogen), b) a 1:1 mixture of 0.5 mg mL^−1^ collagen and 0.4 % low melting agarose, and c) only 0.5 mg mL^−1^ collagen. The agarose hydrogel was prepared in PBS and heated to 65 °C and then brought down to 37 °C for scenario (a) and before mixing it with the collagen for scenario (b). Samples were then prepared in FEP capillaries (Experimental Section) and stored at 37 °C in a humidified atmosphere with 5% CO_2_ for 24 h. Bright‐field images were taken to check for bursts at 24 hpe.

### Statistics

Data noise emerging as part of the image acquisition over a 12 h time series was reduced by applying a median smoothing (median of every five consecutive time points). The application of this smoothing function was mentioned in each figure legend. All error shades (s.e.m.) in plots were generated using an adapted function (shaded area error bar plot, https://www.mathworks.com/matlabcentral/fileexchange/58262‐shaded‐area‐error‐bar‐plot, MATLAB Central File Exchange).

A chi‐squared test was performed to compare between the categories “drug/chemical” (*N* = 10) and “control” (*N* = 3, 10). All other data were tested for significance using the two‐sample independent *t*‐test, considering significance to be *p* ⩽ 0.05. The significant *p*‐values were categorized as *p* > 0.05 = n.s., *p* ⩽ 0.05 = *, *p* ⩽ 0.01 = **, *p* ⩽ 0.0001 = ***, and *p* ⩽ 0.00001 = ****.

## Conflict of Interest

The authors declare no conflict of interest.

## Supporting information

Supporting Information

Supplemental Video 1

Supplemental Video  2

Supplemental Video 3

Supplemental Video 4

Supplemental Video 5

Supplemental Video 6

## Data Availability

Data available on request from the authors.
